# Is praziquantel preventive chemotherapy associated with visual disorders in Eritrea? A comment on the case series reported by Debesai and Russom

**DOI:** 10.1371/journal.pntd.0008827

**Published:** 2020-11-05

**Authors:** Antonio Montresor, Pauline N. Mwinzi, Anthony W. Solomon, Jonathan King, Amadou Garba

**Affiliations:** 1 World Health Organization, Department of Control of Neglected Tropical Diseases, Geneva, Switzerland; 2 World Health Organization, Regional Office for Africa, Brazzaville, Congo; Swiss Tropical and Public Health Institute, SWITZERLAND

Praziquantel is the recommended medicine for preventive chemotherapy (PC) in schistosomiasis-endemic populations [1]. It is also one of the recommended drugs for mass drug administration (MDA) for taeniasis (1). Since coming to market in the 80s, billions of praziquantel doses have been administered worldwide during PC interventions and for treatment of clinical cases. In 2018 alone, more than 95 million people received PC with praziquantel for schistosomiasis [2].

The World Health Organization (WHO) is convinced that it is critical to detect and analyze any signal of adverse events caused by medicines used for clinical or public health purposes. There is a particular imperative to do so for medicines used in PC, in which most recipients are asymptomatic or even uninfected. We therefore welcome the exploratory study by Debesai and Russom [3] who report a potential link between the use of praziquantel and subsequently reported visual disorders.

Visual abnormalities following treatment with praziquantel have not been reported previously but this maybe a result of an insufficient surveillance in place following MDA in other countries. In addition Debesai and Russom [3] report other adverse events that may be consequent to an increase of the intracranial pressure may be an indirect confirmation of cerebral involvement following praziquantel administration.

However, it is difficult to interpret the data presented by Debesai and Russon because limited information is provided on (a) the way in which visual disorders were assessed (by clinical investigation or only by questionnaires administered to praziquantel recipients); (b) the nature of symptoms and/or signs; (c) the extent of resolution of symptoms and/or signs, and how this was evaluated; and (d) the characteristics of the affected individuals (particularly height and weight, to precisely assess the likely doses provided).

We understand that analysis was of data collected into VigiBase, without follow-up of individuals who reported being affected. No controls were included; diseases causing blindness and visual impairment are extremely common in Eritrea [4]. We therefore believe that the methodology only allows identification of a possible association. We suggest caution in labelling this as being “suggestive of a causal association” between praziquantel and visual disorders until further investigation can be completed.

We are also concerned by the statement that the analysis "suggests inaccuracies in the dose of praziquantel, in reference to some African studies, as a possible root cause”, since the dosage provided [5] was not assessed, this suggestion is therefore purely speculative. In addition the references provided [6,7] suggest that the use of the WHO dose pole may results in underdosing and thus do not support the hypothesis of visual impairment as a consequence of praziquantel overdosing.

The WHO praziquantel dose pole has been designed to provide 30–60 mg praziquantel per kilogram body weight, a dose range that is effective against schistosomiasis in a single administration. The recommended dose of praziquantel for neurocysticercosis is of 50 mg/kg for 14 days [8] and is normally well tolerated, in addition Bittencourt et al. [9] reported administration of 100 mg/kg for 10 days to individuals affected by neurocysticercosis without reports of visual impairment.

Assuming that anonymization of data makes tracing previously-affected patients impossible, WHO in collaboration with the Ministry of Health will undertake detailed investigations in Eritrea during the next praziquantel MDA in the same districts where the visual impairment has been reported to (a) actively monitor adverse events associated with praziquantel; (b) evaluate the possible association between praziquantel and visual disorders if they are observed (including through detailed visual and neurologic examination by specialists); and (c) evaluate the performance of the WHO praziquantel dose pole in the Eritrean population, by comparing the doses of praziquantel estimated by the WHO pole and by weighing scales.

Results of those investigations will be shared openly. We continue to welcome comment and collaboration to help ensure the safety of programmes seeking to control, eliminate and eradicate neglected tropical diseases.

## Disclaimer

The authors are staff members of the World Health Organization. The authors alone are responsible for the views expressed in this article and they do not necessarily represent the decisions, policy or views of the World Health Organization.
